# Tuberculosis menace: a case report of disseminated tuberculosis presenting as tubercular meningitis and long-segment cervical tuberculous myelitis in a 32-year-old man from rural India

**DOI:** 10.3389/fmed.2025.1507511

**Published:** 2025-01-30

**Authors:** Saket Satyasham Toshniwal, Jiwan S. Kinkar, Amit Toshniwal, Anshul Sood, Sunil Kumar, Sourya Acharya

**Affiliations:** ^1^Department of General Medicine, Jawaharlal Nehru Medical College, Datta Meghe Institute of Higher Education and Research, Wardha, India; ^2^Department of Neurology, Jawaharlal Nehru Medical College, Datta Meghe Institute of Higher Education and Research, Wardha, India; ^3^Department of Respiratory Medicine, Jawaharlal Nehru Medical College, Datta Meghe Institute of Higher Education and Research, Wardha, India; ^4^Department of Radiology, Jawaharlal Nehru Medical College, Datta Meghe Institute of Higher Education and Research, Wardha, India

**Keywords:** tuberculosis, myelitis, meningitis, disseminated, miliary, hydrocephalus

## Abstract

This case report discusses an uncommon presentation of miliary tuberculosis as tubercular meningitis (TBM) and long-segment cervical tuberculous myelitis in a 32-year-old man from rural India. The patient presented with symptoms of fever, headache, neck stiffness, and gradual weakness in all four limbs. Hydrocephalic changes secondary to meningitis and involvement of the spinal cord were observed on neuroimaging and were correlated with clinical findings of cervical myelitis, confirming the diagnosis of TBM with cervical myelitis. TBM, together with cervical myelitis in patients with miliary tuberculosis (TB), is a rare manifestation in tubercular endemic countries, such as India. It is crucial to confirm the diagnosis and initiate antitubercular therapy (ATT) promptly in order to prevent neurological complications. In this context, the present case highlights the importance of considering TB in patients with neurological manifestations that are not characteristic of the most common diseases. This report also emphasizes the need to raise awareness and improve the management of tuberculosis in rural areas where there are few opportunities to access tertiary centers.

## Introduction

Tuberculosis (TB) is one of the biggest challenges to global healthcare today, especially in developing countries, such as India. It is well known that pulmonary TB is the most common presentation of tuberculosis; however, it can also affect extrapulmonary sites, such as the brain, meninges, and spinal cord, similar to our case (1). Miliary TB occurs when *Mycobacterium tuberculosis* spreads to various body parts including extrapulmonary organs such as the brain, meninges, spinal cord, intestine, lymph nodes, and kidneys. This type of TB poses diagnostic and management challenges due to the lack of specific symptoms. Tubercular meningitis and spinal tuberculosis are less common neurological manifestations of TB. However, if diagnosed late, these conditions can be fatal in many cases (2). This report focuses on the case of a 32-year-old man from a rural area in the central part of India who was diagnosed with both tubercular meningitis (TBM) and long-segment cervical tuberculous myelitis concurrently, which is an unusual finding in clinical practice. The case presented here reflects the need for heightened suspicion regarding TB in endemic regions, particularly concerning the issues emerging in rural practice where access to detailed diagnostic methods and effective treatment is often limited (3).

## Case presentation

A 32-year-old male patient, a laborer by occupation, was brought to the hospital by his relatives in a bedridden and lethargic state with complaints of progressive headache, fever, neck stiffness, and generalized weakness that had persisted for 2 weeks. He also experienced difficulty in walking, numbness, and weakness in both upper and lower limbs. This condition had deteriorated over the preceding days leading up to his confinement to bed.

The patient initially developed an intermittent low-grade fever and a persistent dull headache. Over the course of a week, he developed neck stiffness and photophobia. Approximately 4 days prior to presentation, he experienced worsening weakness in both his upper and lower limbs, which led to difficulty walking, trouble wearing his slippers, and an increasing sense of imbalance. The patient was unable to stand or walk. He could not perform his daily chores—he could neither make a morsel of his food nor hold a glass. He had difficulty lifting his hands above his shoulders. He also reported numbness in his extremities, with reduced sensation in the hands and feet. There was no history of seizures, total loss of consciousness, or trauma. The patient was bedridden and was brought to the hospital by his relatives.

The patient denied any previous history of tuberculosis, human immunodeficiency virus (HIV) infection, or recent immunosuppressive therapy. He had no known contact with individuals diagnosed with tuberculosis and no history of chronic diseases such as diabetes, hypertension, or malignancy. However, during a detailed history-taking session with leading questions, the patient revealed that he had had a low-grade evening fever for more than 2 months, accompanied by a productive, scanty cough and significant weight loss over this period. He also experienced dyspnoea with minimal exertion, which forced him to leave his job.

On general physical examination, the patient was febrile, with a temperature of 38.5°C, lethargic and cachexic, with a pulse rate of 120 bps, regular in rhythm, and normal volume pulse.

On neurological examination, the patient was alert but appeared lethargic, with no focal cranial nerve deficits. Neck stiffness was present with a positive Brudzinski’s sign. On motor system examination, strength was reduced (to one-fifth) in both the upper and lower limbs as the patient was unable to move his extremities. There was significant hypotonia noted in all four limbs, while all deep tendon reflexes were absent with a mute bilateral plantar response—suggestive of acute spinal shock. The patient was catheterized on admission.

His sensory examination could not be assessed as the patient started to get drowsy. A probable diagnosis of acute bacterial meningitis was made, and the patient was immediately shifted to the neuroimaging and intensive care unit for further management.

**Figure 1:**
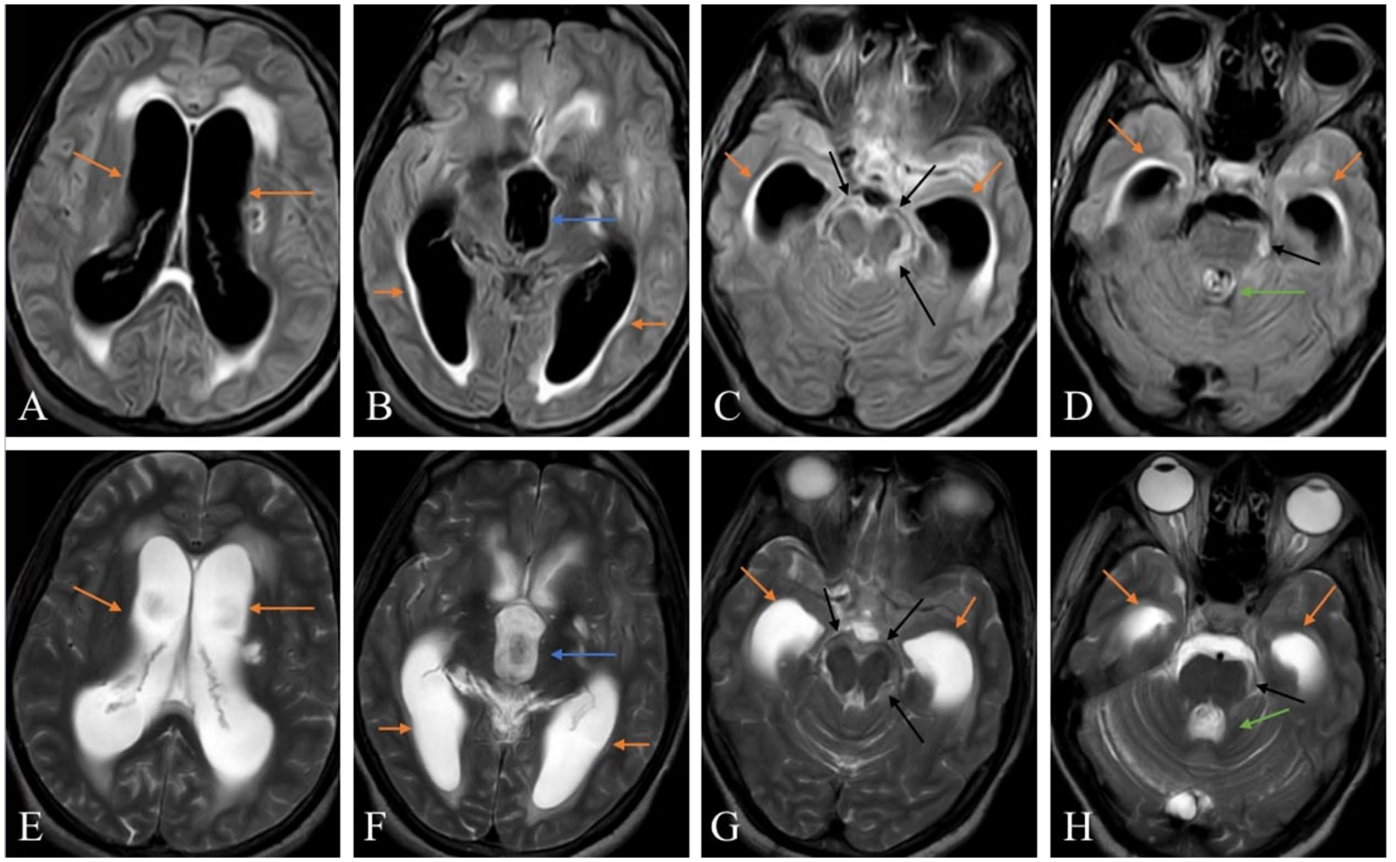
Sequential magnetic resonance imaging (MRI) of the brain fluid attenuation recovery sequence (FLAIR) **(A–D)** and T2-weighted imaging sequence **(E–H)** showing dilated bilateral lateral ventricles (orange arrows), third ventricle (blue arrows) with periventricular oozing and non-dilated fourth ventricle (green arrows) with T2/FLAIR hyperintense debris in the peri-mesencephalic cisterns (black arrows) suggesting extraventricular obstructive hydrocephalus caused by tubercular debris.

Sequential magnetic resonance imaging (MRI) of the brain fluid attenuation recovery sequence (FLAIR) (Figures 1A–D) and T2-weighted imaging sequence (Figures 1E–H) revealed dilated bilateral lateral ventricles and third ventricle with periventricular oozing and a non-dilated fourth ventricle with T2/FLAIR hyperintense debris in the peri-mesencephalic cisterns, suggesting extraventricular obstructive hydrocephalus caused by tubercular debris.

**Figure 2:**
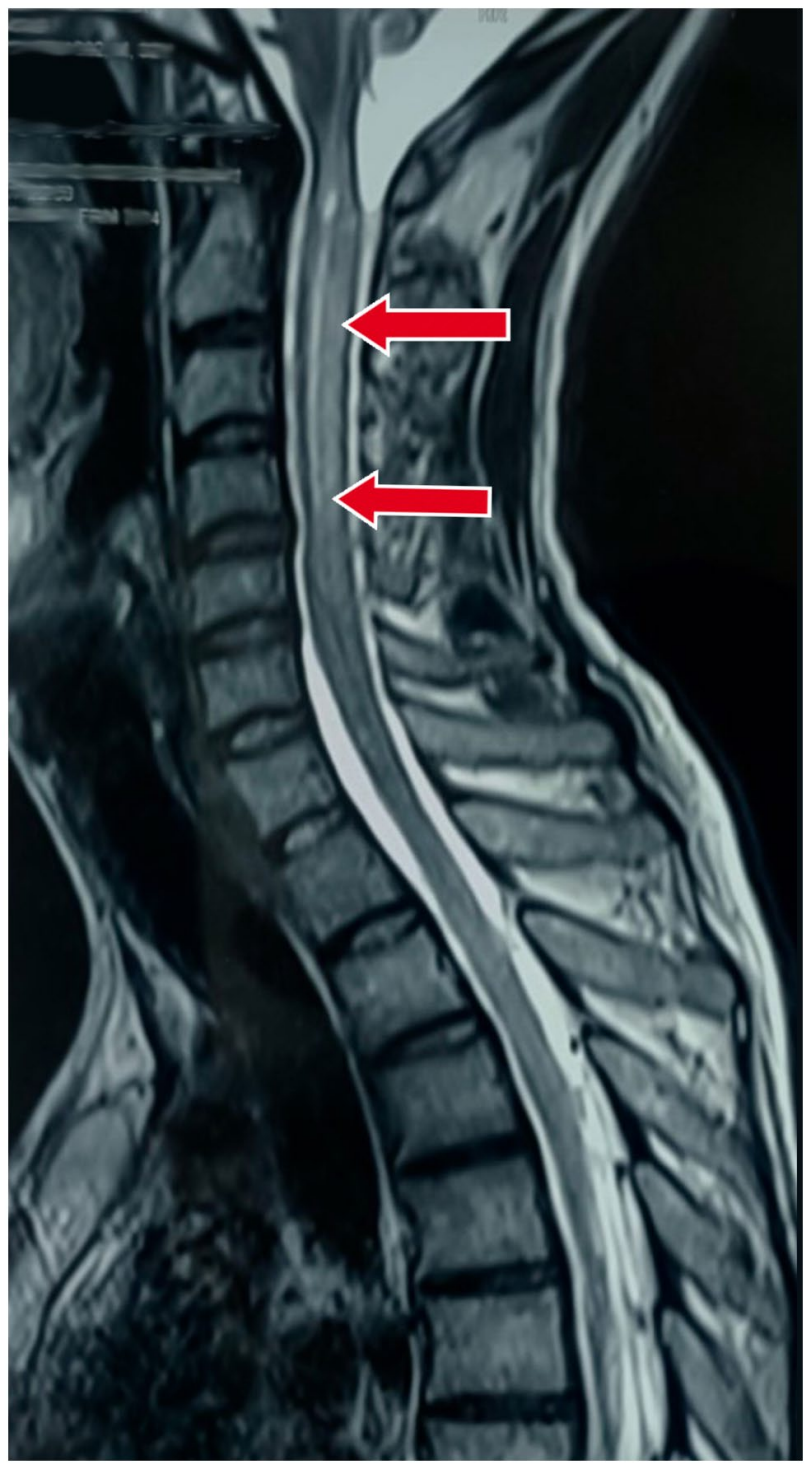
Magnetic resonance imaging (MRI) of the cervical spine in the sagittal section showing longitudinal extensive midline hyperintense lesions on T2-weighted imaging in both the upper and lower cervical spinal cord, suggestive of long-segment cervical cord myelitis with associated cord oedema.

Also, an MRI of the cervical spine with whole spine screening was performed to rule out the cause of the quadriparesis. This revealed longitudinal, extensive midline hyperintense lesions on T2-weighted imaging in both the upper and lower cervical spinal cord, suggestive of long-segment cervical cord myelitis with associated cord oedema as shown in Figure 2.

Further deterioration of neurological status was observed, and ventriculoperitoneal shunting was performed surgically to relieve the raised intracranial pressure.

A respiratory system examination revealed diffuse fine crepitations that were heard bilaterally with decreased air entry.

**Figure 3:**
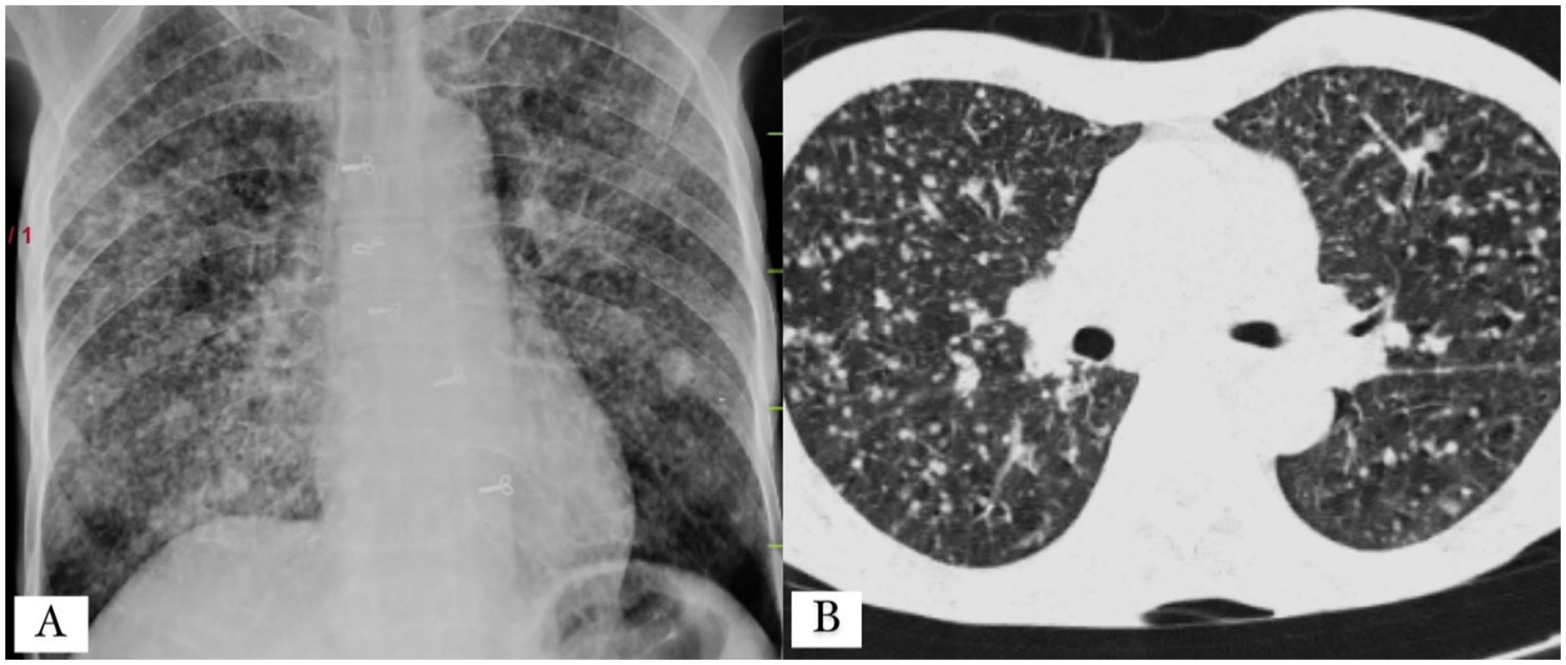
**(A)** Anteroposterior chest X-ray showing multiple homogeneous small nodular opacities in both lung fields, suggesting the presence of miliary mottling. **(B)** High-resolution computed tomography (HRCT) thorax picture showing the snowstorm appearance of miliary mottling as shown in the X-ray.

**Figure 4:**
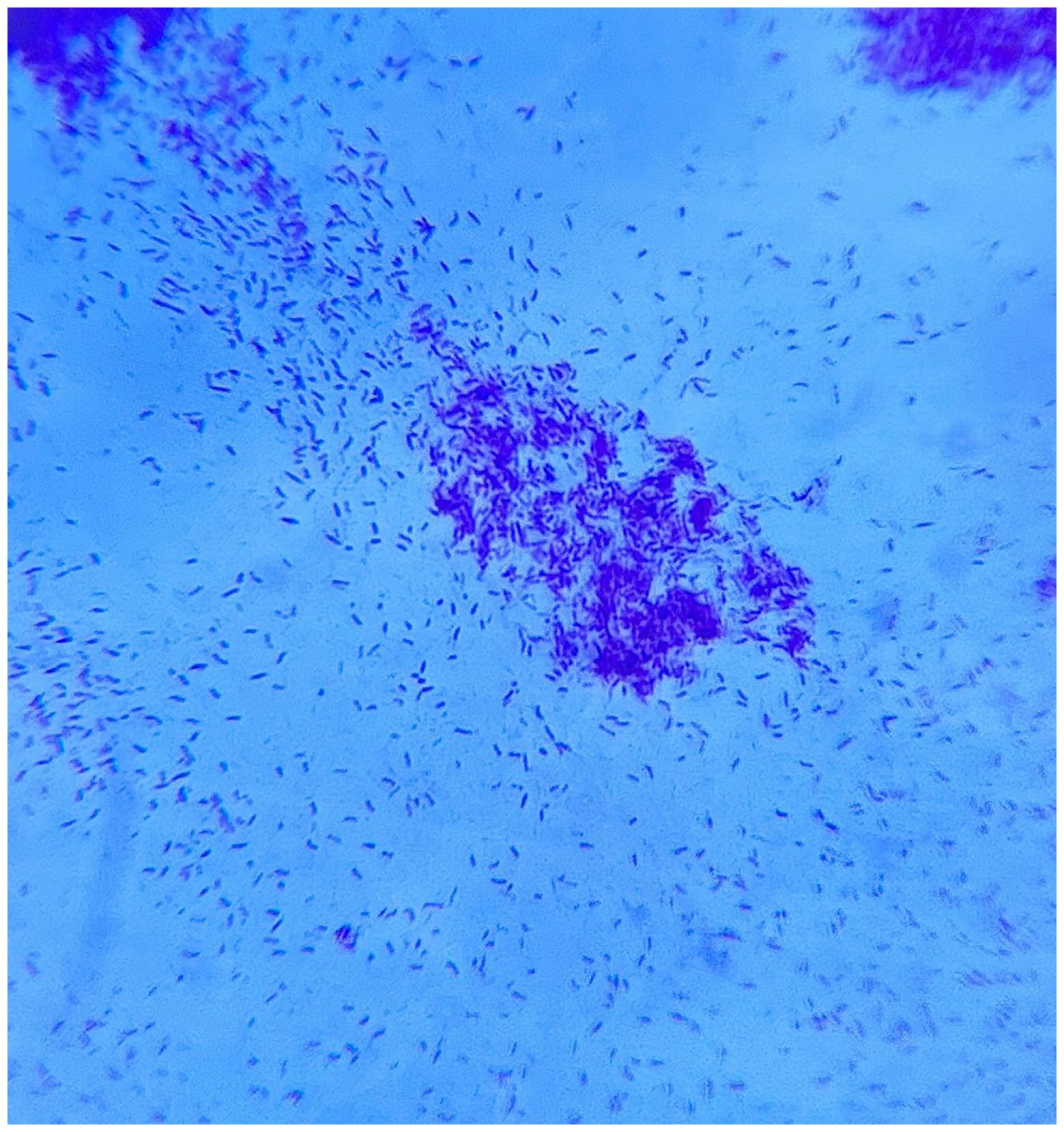
Sputum microscopy showing multiple rod-like bacilli with acid-fast bacilli (AFB) grade-III growth on direct Ziehl Neelsen (ZN) stain microscopy.

A bedside chest X-ray was performed as the history of the patient was highly suggestive of tuberculosis along with a positive respiration system examination, which revealed diffuse fine homogeneous opacities in both lung fields suggestive of miliary mottling as shown in Figure 3A and a high-resolution computed tomography (HRCT) thorax was also performed, which revealed snowstorm appearance of miliary mottling as shown in Figure 3B. Cerebrospinal fluid (CSF) analysis of the patient revealed a clear picture of TBM as listed in Table 1. Multiple rod-like bacilli were present in the sputum Ziehl Neelsen (ZN) stain microscopy with grade-III growth of bacilli, as shown in the microscopic slide picture in Figure 4, which helped confirm the diagnosis of tubercular aetiology of meningitis and tuberculous myelitis, with a positive cartridge-based nucleic acid amplification test (CBNAAT) profile for tuberculosis.

**Table 1:** Cerebrospinal fluid analysis (CSF) of the patient.

CSF parameters	Results
Opening pressure	Elevated
Appearance	Cobweb appearance
Cell count	>500 (predominantly lymphocytes)
Protein	220 mg/dL (elevated)
Glucose	20 mg/dL, low CSF:Blood glucose ratio
ADA	40 mg/dL
CBNAAT	Positive
ZN staining	Negative

ADA, adenosine deaminase; CBNAAT, cartridge-based nucleic acid amplification test; ZN, Ziehl Neelsen.

All other routine laboratory parameters revealed leukocytosis with a normal metabolic panel, raised erythrocyte sedimentation rate, and C-reactive protein with a normal serum iron profile and serum ferritin. Viral marker tests were also performed to rule out any immunocompromised diseases and were negative for HIV, hepatitis C, and hepatitis B. Serum antinuclear antibody (ANA), antibodies for neuromyelitis optica spectrum disorders (NMOSD) and myelin oligodendrocyte (MOG) antibodies were also sent to rule out other causes of long-segment myelitis and were found to be negative.

An MRI of the thoracic and lumbar spine was also performed, which revealed no bony abnormalities or any soft tissue involvement ruling out the possibility of Pott’s tuberculosis spine.

Owing to the history and investigatory findings mentioned above, a diagnosis of TB meningitis with TB myelitis with disseminated tuberculosis was made.

The patient was started on anti-tubercular therapy (ATT), which included the following medications:

- Isoniazid (INH): 300 mg/day.
- Rifampicin: 600 mg/day.
- Pyrazinamide: 1,500 mg/day.
- Ethambutol: 1,200 mg/day.

Additionally, an injection of streptomycin was administered due to the extensive central nervous system involvement of tuberculosis.

The patient was also started on intravenous dexamethasone to reduce spinal cord inflammation and prevent further damage. Prophylactic intravenous antiepileptics were also started as the patient was prone to seizures.

The patient was closely monitored for response to therapy. Over the course of a week, improvement was observed in the patient’s fever, neck stiffness, and sensorium, along with a slight improvement in the strength of all four limbs. The patient was eventually shifted to the ward. Rigorous physiotherapy and rehabilitation treatment were started. Improvement in the patient’s neurological and motor abilities was monitored. However, the patient required a prolonged course of steroid treatment lasting 6 weeks, followed by a tapering dosage protocol, in addition to continuing the ATT for 12 months.

During subsequent follow-up, the patient’s condition improved with no recurrence of neurological symptoms or deterioration in neurological status, and he was advised to complete the treatment course with close follow-up.

A summarizing sequence of events with relevant data of this case is explained with a listing in Table 2.

**Table 2:** Summarizing events and timeline of relevant case report data.

Sequential events of the case	Interpretation with relevant data
1. Case presentation (symptoms and brief history)	A 32-year-old male patient presented with complaints of progressive headache, fever, neck stiffness, and generalized weakness that had persisted for 2 weeks and weakness in both upper and lower limbs, which had worsened over the preceding days to the point of bedridden status. The patient also had a history of low-grade evening rise fever for more than 2 months, associated with productive scanty cough and significant weight loss over a span of 2 months. Past history was non significant.
2. Clinical examination (general and systemic examination)	General examination: Febrile, lethargic, and cachexic, tachycardia with tachypnoea. On neurological examination, the patient was alert but drowsy. Brudzinski’s and Kernig’s signs were positive. On motor system examination, strength was reduced (to one-fifth) in both upper and lower limbs, with hypotonia noted in all four limbs, while all deep tendon reflexes were absent with a mute bilateral plantar response—suggestive of acute spinal shock. On respiratory examination, there was decreased air entry bilaterally with diffuse fine crepitations over both lung fields.
3. Investigations	MRI of the brain: Meningeal enhancement with extraventricular obstructive hydrocephalus with hyperintense debris on the basal cisterna (Figures 1A–H) MRI of the cervical spine: Longitudinal extensive hyperintense lesions predominantly in the upper cervical and the lower cervical regions (Figure 2). CSF analysis: Cobweb appearance with elevated pressure, proteins, and cells (predominantly lymphocytes) with positive CBNAAT and raised ADA (Table 1). Chest X-ray: Multiple homogeneous small nodular opacities are present in both lung fields suggesting miliary mottling (Figure 3A) HRCT thorax: Snowstorm appearance of miliary mottling (Figure 3B) Sputum examination: Acid-fast bacilli (AFB) grade-III growth on direct Ziehl Neelsen (ZN) stain microscopy (Figure 4). Routine investigations: Within normal limits with non-reactive tests for HIV, hepatitis B, and hepatitis C. Autoimmune profile: Negative ANA with negative for NMOSD and multiple sclerosis.
4. Diagnosis	Tubercular meningitis (TBM) with upper cervical tuberculous myelitis.
5. Treatment	Anti-tubercular therapy for 12 months with steroids for 6 weeks.
6. Outcome and follow-up	Gradual improvement in sensorium and other neurological deficits. Total improvement in strength on subsequent follow-up with no focal neurological deficits.

## Discussion

Tubercular meningitis (TBM) is a severe, life-threatening condition caused by *M. tuberculosis* that invades the central nervous system (CNS), primarily affecting the meninges that surround the brain and the spinal cord (4).

The co-occurrence of miliary tuberculosis and tubercular meningitis is well documented in the literature. A study by Wang et al. highlighted the high frequency of CNS involvement in miliary TB, occurring in over 60% of cases in their cohort, with TBM being a prominent manifestation. This finding underscores the hematogenous dissemination pathway of *M. tuberculosis*, which can lead to multi-organ involvement, including the CNS (5). The bacteria form small tubercles in the meninges, subarachnoid spaces, or ventricular walls (known as a Rich focus). When these tubercles rupture into the subarachnoid space, it leads to inflammation, thickening of the meninges, vasculitis, and exudative changes, which can lead to cranial nerve palsies, hydrocephalus, and infarction (5).

Tuberculous myelitis—an uncommon manifestation of CNS tuberculosis—affects the spinal cord; the infection can occur through direct extension via the meninges or through haematogenous spread (6). It can present as multiple lesions or can be focal. Additionally, it can also present with longitudinal extensive spinal cord lesions involving more than one segment of the spinal cord, as noted in this case.

This type of myelitis frequently exhibits inflammatory alterations in the spinal cord, resulting in demyelination and axonal damage. As a result, neurological impairments may occur, including weakness, sensory loss, and bladder dysfunction. The inflammation in severe cases can lead to spinal cord infarction, which can result in effects that could be irreversible (6).

As discussed in this article, neurological complications are common in TBM and can lead to significant disability when the spinal cord is affected. Early identification of these complications is essential in order to prevent irreversible neurological damage.

One of the key challenges in diagnosing TBM, when spinal cord involvement is present, is the non-specific nature of the symptoms. Fever, headache, and neck stiffness are symptoms common to many infectious and non-infectious types of meningitis (7). In this case, the presence of additional symptoms of progressive limb weakness, numbness, and difficulty walking prompted further investigation into potential spinal cord involvement.

MRI remains the gold standard for diagnosing meningeal and spinal cord involvement in tuberculosis. In this patient, MRI findings of diffuse meningeal enhancement and longitudinal spinal cord lesions were crucial in establishing the diagnosis (8). Additionally, high-resolution computed tomography (CT) of the thorax revealed a miliary pattern, indicating disseminated TB, which further supported the likelihood of CNS involvement.

With its characteristic findings of elevated protein levels, low glucose levels, and lymphocytic pleocytosis, CSF analysis is an essential diagnostic tool in TBM. The presence of acid-fast bacilli in the CSF confirmed the diagnosis, although the CSF culture results were pending at the time of this report. Despite the availability of these diagnostic tools, delays in diagnosis are common, particularly in resource-limited settings where access to advanced imaging and laboratory tests may be limited (9).

The mainstay of treatment for tubercular meningitis and tuberculous myelitis is a prolonged course of antitubercular therapy (ATT). In this case, the patient was started on the standard four-drug regimen consisting of isoniazid, rifampicin, pyrazinamide, and ethambutol. The duration of treatment for CNS tuberculosis is typically longer than that for pulmonary tuberculosis, often extending to 9–12 months or more, depending on the clinical and radiological response to therapy (10).

Steroids have been shown to improve outcomes in TBM by reducing the risk of death and disabling neurological complications, particularly in patients with severe disease or extensive CNS involvement (10).

Close monitoring of the patient’s clinical status, repeated imaging, and CSF analysis are essential to assess the response to therapy. Particularly, a follow-up MRI of the spine will help determine the extent of resolution of the spinal cord lesions. At the same time, repeat CSF analysis can provide insight into the ongoing inflammatory response and the effectiveness of the treatment regimen (11).

The overall outcome of TBM with spinal cord involvement primarily reflects the severity of neurological deficits at the time of diagnosis and the delay in initiating appropriate treatment. In this patient, both TBM and tuberculous myelitis were identified early, and appropriate ATT and corticosteroids were started before the neurological health of the patient deteriorated any further. There is a potential for regaining neurological functions—especially in situations complicating spinal cord involvement—albeit at a slow pace with only partial success even with the relevant treatment (12).

Residual neurological abnormalities that manifest several months or years after tuberculous myelitis include motor paralysis, peripheral neuropathy, and urinary incontinence. The extent of the recovery is directly associated with the degree of spinal cord inflammation and the effectiveness of appropriate treatment. Subsequently, neurological examinations, imaging, and functional rehabilitation need to be performed regularly to support the patient’s recovery (13).

The co-occurrence of tubercular meningitis and long-segment cervical tuberculous myelitis in the same patient is an uncommon manifestation of disseminated tuberculosis. In addition, the involvement of a long cervical spinal cord segment is a rare manifestation of tuberculous myelitis, requiring meticulous differentiation from other aetiologies, such as autoimmune, neoplastic, or vascular disease (13). This dual CNS involvement presents unique diagnostic and therapeutic challenges that are rarely documented in patients from rural or resource-constrained settings. Moreover, this case serves as a stark reminder of the intersection of tuberculosis burden and healthcare disparities in rural settings, emphasizing the need for systemic improvements in TB diagnosis and management and necessitating a high index of clinical suspicion for prompt diagnosis and treatment.

## Conclusion

This case report highlights the complexity of disseminated tuberculosis involving both the brain and the spinal cord. Tubercular meningitis, when accompanied by tuberculous myelitis, represents a severe form of CNS tuberculosis that requires prompt recognition and aggressive treatment. Early diagnosis using MRI and CSF analysis, followed by appropriate anti-tubercular therapy and corticosteroids, is critical to improving outcomes in these patients. This case underscores the importance of maintaining a high index of suspicion for spinal cord involvement in patients who present with neurological symptoms in the context of TBM, as early intervention can prevent irreversible neurological damage and improve long-term prognosis.

## Data Availability

The original contributions presented in the study are included in the article/supplementary material, further inquiries can be directed to the corresponding author.
